# Unrecognized implementation science engagement among health researchers in the USA: a national survey

**DOI:** 10.1186/s43058-020-00027-3

**Published:** 2020-03-30

**Authors:** Elizabeth R. Stevens, Donna Shelley, Bernadette Boden-Albala

**Affiliations:** 1 Department of Population Health, NYU Langone Health, New York, NY USA; 2 College of Global Public Health, NYU, New York, NY USA; 3 Susan and Henry Samueli College of Health Sciences, UC Irvine, Irvine, CA USA

**Keywords:** Implementation science, Health research, Unrecognized, Engagement, Awareness

## Abstract

**Background:**

Implementation science (IS) has the potential to serve an important role in encouraging the successful uptake of evidence-based interventions. The current state of IS awareness and engagement among health researchers, however, is relatively unknown.

**Methods:**

To determine IS awareness and engagement among health researchers, we performed an online survey of health researchers in the USA in 2018. Basic science researchers were excluded from the sample. Engagement in and awareness of IS were measured with multiple questionnaire items that both directly and indirectly ask about IS methods used. Unrecognized IS engagement was defined as participating in research using IS elements and not indicating IS as a research method used. We performed simple logistic regressions and tested multivariable logistic regression models of researcher characteristics as predictors of IS engagement.

**Results:**

Of the 1767 health researchers who completed the survey, 68% stated they would be able to describe IS. Only 12.7% of the population self-identified as using IS methods. Of the researchers not self-identifying as using IS methods, 86.4% reported using the IS elements “at least some of the time.” Nearly half (47.9%) reported using process/implementation evaluation, 89.2% use IS measures, 27.3% use IS frameworks, and 75.6% investigate or examine ways to integrate interventions into routine health settings. IS awareness significantly reduced the likelihood of all measures of unrecognized IS engagement (aOR 0.13, 95% CI 0.07 to 0.27, *p* < 0.001).

**Conclusion:**

Overall, awareness of IS is high among health researchers, yet there is also a high prevalence of unrecognized IS engagement. Efforts are needed to further disseminate what constitutes IS research and increase IS awareness among health researchers.

Contributions to the literature

- More researchers are beginning to incorporate implementation concepts into their research.
- Using implementation elements without knowing they are part of the field of implementation science (IS) may jeopardize the rigor of the research.
- There is a high prevalence of researchers engaging in implementation research without recognizing their methods as IS.
- Efforts are needed to further disseminate what constitutes IS research and increase IS awareness among health researchers.

Contributions to the literature

- More researchers are beginning to incorporate implementation concepts into their research.
- Using implementation elements without knowing they are part of the field of implementation science (IS) may jeopardize the rigor of the research.
- There is a high prevalence of researchers engaging in implementation research without recognizing their methods as IS.
- Efforts are needed to further disseminate what constitutes IS research and increase IS awareness among health researchers.

## Background

Over the past 15 years, as a field, implementation science (IS) has made great strides to raise awareness of IS as well as establish methods and frameworks that provide for rigorous and meaningful implementation research [1, 2]. Defined as “the scientific study of methods to promote the systematic uptake of research findings and other evidence-based practices into routine practice and, hence, to improve the quality and effectiveness of health services” [3], the appropriate and rigorous use of IS can promote the dissemination and increase the effectiveness of interventions in real-world settings [4, 5].

As efforts have emerged to advance IS, resources have been developed seeking to increase awareness of the importance of the field, as well as develop an understanding of the qualities that make an implementation study rigorous and “good” [1, 2, 6]. The scope of IS is broad, and can be challenging for uninitiated investigators to define, as it encompasses a range of methods both unique to IS as well as derived from other disciplines [1]. Therefore, the broad scope of IS can make it difficult to identify and differentiate from other types of research.

More researchers are beginning to incorporate implementation concepts into their research. Indeed, many non-IS research funding opportunities now expect the incorporation of components of implementation into funding proposals [7]. However, using implementation elements without an awareness that they are part of an established set of methods may jeopardize the rigor of the implementation research performed. Further, while not all researchers are expected to become IS experts, a lack of awareness of IS methods may impede collaboration between researchers during implementation-focused research [8]. As a result, ensuring researchers who engage in implementation research are aware of IS methods included in their research is pivotal to impactful implementation research.

As the field of IS advances, the engagement and collaboration of health researchers across disciplines will serve an important role in the successful implementation of evidence-based interventions. Current levels of IS engagement and the use of implementation methods among health researchers are not clear. To address this knowledge gap, we performed a survey of health researchers to measure awareness of and engagement in IS research.

## Methods

### Participants

The survey was distributed from January to March 2018 by e-mail to health researchers who received federal funding (including all NIH institutes, as well as the Centers for Disease Control and Prevention [CDC], Agency for Healthcare Research and Quality [AHRQ], Health Resources and Services Administration [HRSA], Administration for Children and Family [ACF], and U.S. Department of Veterans Affairs [VA], but not including Patient-Centered Outcomes Research Institute [PCORI]) in the past 5 years. Basic science researchers and non-research grant recipients (e.g., small business grants or conference grants) were excluded from the sample. The sampling frame consisted of participant e-mails obtained from NIH RePORTER [9]. A simple random sample of researchers received the survey. The New York University institutional review board approved the study.

### Survey

Data were collected via an online questionnaire examining participant demographics, current research practices, and perceptions of IS. Survey item development was guided by expert opinion and behavioral models [10–12]. The survey questions were pilot tested with a sample of health researchers from a variety of fields. The survey collected quantitative data including responses on a Likert scale and categorical responses. The survey was distributed by email via Qualtrics, and all responses were anonymous. The relevant survey measures can be found in the appendix.

### Defining engagement in implementation science

Engagement in IS was measured with multiple questionnaire items both directly and indirectly asking about the use of IS methods. Measures of IS engagement included using an IS framework; performing a process/implementation evaluation; research integrating an intervention into routine settings; incorporating measures of acceptability, adoption, appropriateness, feasibility, fidelity, implementation cost, penetration, and sustainability into existing research design. A researcher was considered to have performed IS elements if they indicated that they performed one or more IS elements at least “sometimes,” they report performing or collaborating on an implementation study in the past five years, and/or they self-identified as using “implementation science” methods. This approach for defining engagement was chosen because researchers may not be familiar with IS terminology and methods even if they are using elements of IS in their research. A cut-off of “sometimes” was selected to capture researchers using IS elements even if IS does not represent the majority of the research they engage in. Participants were asked to report the methods they use in their research from a list of research methods, researchers could select multiple methods. Unrecognized IS engagement was thus defined as participating in research using an IS element and not indicating IS as a research method used. Similarly, IS awareness was assessed by asking whether they would be able to describe IS to a colleague. All measures were self-report. See supplementary materials for the text of survey questions pertaining to IS engagement.

### Data analysis

Surveys of less than 85% complete were excluded from analyses. All surveys were examined for inconsistencies and invalid responses were treated as missing values, resulting in slightly different denominators for analyses. We performed descriptive data analysis and multivariable logistic regressions, controlling for the participant demographics, to compare the characteristics of health researchers who use IS to those who do not report its use and assess which researcher characteristics are associated with unrecognized IS engagement. Results are reported as adjusted odds ratios (aOR) with 95% confidence intervals (95% CI). All analyses were performed in Stata (version 14, College Station, TX).

## Results

### Participant characteristics

The survey was distributed to 7259 health researchers. Nearly 30% (2051) of participants started the survey and 1767 participants completed at least 85% of the survey for an overall response rate of 24.3%. The population of non-completers differed significantly from those who completed the survey. Compared to survey completers, non-completers were more likely to only have a master’s degree (6.1% vs. 3.5%), less likely to self-identify as using IS methods (3.8% vs. 12.7%), less likely to report RCTs (22.3% vs. 43.0%), cohort studies (20.3% vs. 29.4%), and epidemiology (12.6% vs. 23.8%) as a method used.

The characteristics of respondents who completed the survey are presented in Table 1. Respondent demographics were generally representative of the NIH funded population [13]. Participants were geographically diverse within the USA institution types included academic (87.4%), public (19.1%), non-profit (14.0%), and private (3.4%). As their highest degree received, 69.4% had a PhD alone, 12.3% had an MD and master’s degree, 9.9% had an MD alone, and 3.6% and 3.5% had an MD-PhD or master’s degree alone, respectively. The most common reported research methods were RCTs (43.0%), cohort studies (29.4%), and epidemiology (23.8%), qualitative research (17.9%), and statistics (17.5%). IS method use was reported by 12.7% of participants.

**Table 1:** Demographic and research characteristics of the study population

	No. participants	%
Demographics	(*n* = 1767)	
Age (years)		
< 35	120	6.8
35–44	450	25.5
45–54	453	25.7
55–64	454	25.8
65+	285	16.2
Sex		
Male	807	45.7
Female	954	54.0
Race		
White	1444	82.4
Black	54	3.1
Hispanic	52	3.0
Asian	138	7.9
Other	26	1.5
Education		
PhD	1218	69.4
MD	174	9.9
MD, PhD	64	3.6
MD, Masters	219	12.5
Masters	62	3.5
Region		
Northeast	485	27.5
Midwest	360	20.4
South	497	28.2
West	365	20.7
International	35	2.0
Years in health research		
< 1 to 10	332	18.8
11 to 20	655	37.1
21 to 30	410	23.2
31 or more	369	20.9
Institution type^a^		
Academic	1544	87.4
Public	337	19.1
Non-profit	248	14.0
Private	60	3.4
Other	9	0.5
Funding type^a^		
Public	1689	95.6
Foundation	275	15.6
Private	103	5.8
Other	83	4.7
Clinical and Translational Science Award (CTSA)		
Yes	1084	61.3
No	383	21.7
Methods used*		
Implementation science	223	12.7
RCTs	759	43.0
Cohort studies	518	29.4
Epidemiology	419	23.8
Population studies	272	15.5
Statistics	308	17.5
Qualitative	315	17.9
Other	1025	58.3

*RCTs* randomized controlled trials

^a^Respondents could select multiple responses therefore values can sum to greater than 100%

### Implementation science awareness and engagement

Although only 12.7% of the population self-identified as using IS methods, 93.8% reported at least sometimes using elements of IS (Table 2). Of the researchers not identifying as using IS methods, 86.4% at least sometimes use elements of IS in their research. Nearly half (47.9%) of researchers reported using process/implementation evaluations, 89.2% reported using IS measures, 27.3% reported using IS frameworks, and 75.6% reported developing or testing ways to integrate interventions into routine health settings. More than two-fifths (43.7%) of respondents reported they performed or collaborated on a study examining the translation of an intervention into routine settings in the past five years. Nearly two-thirds (63.9%) of researchers not self-identifying as using IS methods stated they would be able to describe IS to a colleague.

**Table 2:** Implementation science element performance by self-reported IS methods use

	Overall		Stated IS method use				
			Yes		No		
	No. of participants	%	No. of participants	%	No. of participants	%	*p* value
	(*n* = 1767)		(*n* = 223)		(*n* = 1536)		
Can describe IS	1201	68.0	214	96.0	982	63.9	< 0.001
Perform process evaluation	944	53.4	208	93.3	733	47.9	< 0.001
Use IS measures	1591	90.3	221	99.1	1365	89.2	< 0.001
Use IS frameworks	514	29.2	96	43.0	417	27.3	< 0.001
Develop/test way to integrate interventions into routine health settings	1377	78.2	217	97.3	1156	75.6	< 0.001
Performed/collaborated examining intervention translation into routine settings	869	49.7	204	91.9	664	43.7	< 0.001

*IS* implementation science

### Characteristics associated with unrecognized IS engagement

Researcher characteristics associated with unrecognized IS engagement are presented in Table 3. IS awareness significantly reduced the likelihood of all measures of unrecognized IS engagement (aOR 0.13, 95% CI 0.07 to 0.27, *p* < 0.001). IS awareness decreased unrecognized process/implementation evaluations (aOR 0.14, 95% CI 0.06 to 0.33, *p* < 0.001), use of IS measures (aOR 0.14, 95% CI 0.07 to 0.29, *p* < 0.001), use of IS frameworks (aOR 0.23, 95% CI 0.08 to 0.68, *p* < 0.001), and research integrating an intervention into routine settings (aOR 0.16, 95% CI 0.08 to 0.33, *p* < 0.001).

**Table 3:** Researcher characteristics associated with unrecognized IS engagement

Unrecognized IS element	All IS engagement				Process evaluations				IS measures				IS frameworks				Integrating interventions into routine settings			
Characteristic	%	aOR	95% CI	*p* value	%	aOR	95% CI	*p* value	%	aOR	95% CI	*p* value	%	aOR	95% CI	*p* value	%	aOR	95% CI	*p* value
Age (years)																				
< 35	92.2	1.24	0.39 to 3.92	0.709	80.4	0.92	0.27 to 3.16	0.893	91.8	1.14	0.36 to 3.62	0.822	85.2	1.35	0.21 to 8.80	0.753	90.7	1.13	0.35 to 3.64	0.837
35–44	87.5	1.54	0.70 to 3.39	0.279	76.3	1.14	0.48 to 2.70	0.773	87.1	1.42	0.64 to 3.13	0.387	82.8	1.27	0.36 to 4.49	0.707	85.4	1.54	0.69 to 3.42	0.293
45–54	82.9	0.72	0.36 to 1.46	0.366	75.2	0.78	0.36 to 1.68	0.526	81.9	0.66	0.33 to 1.34	0.251	76.4	0.67	0.22 to 2.03	0.480	80.4	0.75	0.37 to 1.53	0.429
55–64	87.1	0.98	0.54 to 1.78	0.948	80.6	1.07	0.55 to 2.06	0.842	87.0	0.93	0.51 to 1.70	0.819	81.6	0.95	0.36 to 2.52	0.919	85.2	1.06	0.58 to 1.94	0.847
65+	87.3	0.67	0.96 to 56.89	0.054	79.7	1			87.3	1			85.4	1			84.2	1		
Sex																				
Female	85.1	1.02	0.70 to 1.48	0.919	74.9	0.89	0.6 to 1.33	0.577	84.7	0.99	0.68 to 1.44	0.938	79.2	0.99	0.54 to 1.83	0.977	82.9	1.01	0.69 to 1.48	0.967
Male	88.0	1			81.3	1			87.6	1			83.5	1			85.8	1		
Race																				
White	86.7	7.41	0.96 to 56.89	0.054	77.9	1			86.3	1			81.4	1			84.5	1		
Black	78.4	0.53	0.30 to 0.94	0.031	67.7	0.64	0.25 to 1.66	0.357	77.6	0.67	0.29 to 1.56	0.354	77.8	0.85	0.08 to 9.47	0.893	76.1	0.67	0.29 to 1.56	0.353
Hispanic	95.9	2.38	0.29 to 19.85	0.423	93.1	8.56	1.06 to 68.93	0.044	95.7	7.03	0.91 to 54.2	0.061	100	–	–	–	95.2	8.09	1.05 to 62.34	0.045
Asian	83.5	0.77	0.47 to 1.26	0.293	75.0	0.65	0.34 to 1.23	0.183	82.4	0.51	0.29 to 0.92	0.024	72.2	0.35	0.13 to 0.98	0.046	80.2	0.47	0.26 to 0.85	0.013
Other	92.3	0.83	0.51 to 1.33	0.437	88.2	2.74	0.31 to 24.19	0.365	91.3	2.22	0.26 to 18.97	0.468	88.9	2.02	0.19 to 21.91	0.562	91.3	2.26	0.27 to 18.98	0.452
Education																				
PhD	87.2	0.96	1			1			86.8	1			81.3	1			84.9	1		
MD	86.2	0.93	0.40 to 2.14	0.863	78.9	0.72	0.37 to 1.40	0.337	85.6	0.73	0.4 to 1.33	0.306	80.4	0.70	0.27 to 1.81	0.464	83.8	0.75	0.41 to 1.38	0.359
MD, PhD	86.0	0.89	0.45 to 1.75	0.731	74.2	0.59	0.22 to 1.61	0.305	85.7	0.81	0.33 to 2.01	0.656	70.0	0.51	0.16 to 1.61	0.250	85.7	0.99	0.38 to 2.60	0.985
MD, Masters	80.4	1.17	0.63 to 2.15	0.619	72.8	0.85	0.51 to 1.42	0.546	80.3	0.85	0.53 to 1.37	0.510	76.3	0.82	0.36 to 1.85	0.633	78.5	0.84	0.51 to 1.36	0.469
Masters	88.1	0.54	0.36 to 0.81	< 0.01	80.6	1.26	0.37 to 4.28	0.709	87.7	0.99	0.3 to 3.26	0.984	90.0	0.71	0.10 to 4.86	0.727	86.8	1.10	0.33 to 3.64	0.880
Region																				
Northeast	88.2	0.83	0.50 to 1.38	0.470	81.4	1			88.1	1			85.2	1			86.1	1		
Midwest	84.2	0.59	0.14 to 2.46	0.467	73.3	0.68	0.39 to 1.18	0.167	83.5	0.75	0.46 to 1.23	0.254	75.2	0.74	0.32 to 1.72	0.483	81.5	0.76	0.46 to 1.25	0.277
South	87.5	0.75	0.42 to 1.36	0.349	79.7	0.80	0.48 to 1.33	0.388	87.0	0.82	0.51 to 1.33	0.423	81.1	0.70	0.32 to 1.50	0.358	84.7	0.77	0.47 to 1.26	0.299
West	86.1	0.80	0.33 to 1.97	0.628	77.5	0.84	0.48 to 1.48	0.546	85.9	0.85	0.51 to 1.42	0.528	82.4	0.70	0.30 to 1.62	0.402	84.9	0.87	0.51 to 1.47	0.605
International	76.5	0.82	0.51 to 1.31	0.398	61.9	0.40	0.08 to 2.05	0.274	74.2	0.52	0.12 to 2.29	0.388	71.4	0.47	0.03 to 6.63	0.578	72.4	0.53	0.12 to 2.30	0.394
Years in health research																				
< 1 to 10	88.9	0.93	0.40 to 2.14	0.863	80.8	1.05	0.43 to 2.58	0.919	88.5	0.93	0.40 to 2.15	0.864	82.6	0.59	0.15 to 2.23	0.433	87.5	1.11	0.47 to 2.63	0.805
11 to 20	84.3	0.89	0.45 to 1.75	0.731	73.7	0.85	0.41 to 1.77	0.665	83.8	0.91	0.46 to 1.81	0.798	79.2	0.66	0.23 to 1.88	0.439	81.7	1.01	0.51 to 2.02	0.970
21 to 30	86.8	1.17	0.63 to 2.15	0.619	78.8	0.98	0.50 to 1.90	0.951	86.3	1.17	0.63 to 2.16	0.618	79.2	0.76	0.28 to 2.06	0.596	84.6	1.27	0.68 to 2.36	0.458
31 or more	87.9	1			82.1	1			87.8	1			86.4	1			85.3	1		
Institution type*																				
Academic	86.5	1			77.1	1			90.1	1			29.2	1			77.8	1		
Public	80.3	0.54	0.36 to 0.81	< 0.01	72.2	0.62	0.40 to 0.97	0.034	91.0	0.54	0.36 to 0.81	< 0.01	28.7	0.57	0.29 to 1.12	0.100	82.3	0.54	0.36 to 0.82	< 0.01
Non-profit	87.8	1.21	0.73 to 2.02	0.454	81.8	1.26	0.73 to 2.18	0.415	90.7	1.15	0.69 to 1.91	0.597	29.1	2.93	1.05 to 8.18	0.041	78.1	1.15	0.68 to 1.92	0.602
Private	84.7	0.74	0.31 to 1.78	0.500	71.9	0.57	0.21 to 1.51	0.254	91.7	0.73	0.30 to 1.76	0.483	25.0	0.70	0.14 to 3.36	0.652	71.7	0.59	0.24 to 1.45	0.249
Funding type*																				
Public	86.1	1			77.4	1			90.4	1			29.4	1			78.5	1		
Foundation	80.2	0.64	0.42 to 0.98	0.039	70.7	0.68	0.43 to 1.08	0.099	92.0	0.62	0.40 to 0.95	0.027	32.8	0.78	0.39 to 1.56	0.484	83.9	0.66	0.43 to 1.03	0.067
Private	87.6	1.11	0.52 to 2.37	0.787	81.0	1.11	0.48 to 2.55	0.808	88.2	1.08	0.51 to 2.32	0.834	33.3	0.67	0.22 to 2.07	0.486	80.4	1.15	0.54 to 2.47	0.718
Other	92.1	2.98	0.99 to 8.99	0.053	87.0	2.93	0.93 to 9.26	0.067	92.7	3.15	1.04 to 9.53	0.043	15.9	1.92	0.18 to 20.16	0.588	79.3	3.13	1.04 to 9.45	0.043
CTSI																				
Yes	85.2	0.78	0.50 to 1.22	0.277	75.8	0.77	0.48 to 1.23	0.275	84.9	0.79	0.50 to 1.24	0.299	77.9	0.51	0.24 to 1.09	0.085	82.8	0.74	0.47 to 1.17	0.199
No	88.6	1			82.9	1			88.1	1			87.6	1			86.9	1		
Methods used*																				
RCTs	83.1	0.66	0.46 to 0.93	0.018	75.4	0.73	0.50 to 1.08	0.115	94.1	0.65	0.46 to 0.93	0.018	35.0	0.64	0.36 to 1.15	0.132	88.5	0.73	0.51 to 1.04	0.084
Cohort	87.6	1.26	0.85 to 1.86	0.246	77.6	1.12	0.72 to 1.73	0.609	93.8	1.24	0.84 to 1.84	0.275	30.0	1.53	0.81 to 2.88	0.191	82.6	1.29	0.86 to 1.91	0.217
Epidemiology	86.3	1			76.1	1			95.2	1			30.2	1			81.3	1		
Pop. studies	84.1	1.08	0.68 to 1.69	0.751	76.7	1.21	0.74 to 1.99	0.453	93.8	1.06	0.67 to 1.67	0.805	35.3	1.18	0.56 to 2.50	0.658	80.9	1.06	0.66 to 1.69	0.805
Statistics	86	0.98	0.61 to 1.57	0.927	75.7	1.05	0.62 to 1.77	0.856	88.3	0.95	0.59 to 1.52	0.826	22.7	1.19	0.5 to 2.82	0.701	71.4	1.01	0.62 to 1.64	0.983
Qualitative	69.2	0.24	0.16 to 0.36	< 0.001	60.0	0.30	0.20 to 0.46	< 0.001	96.8	0.24	0.16 to 0.36	< 0.001	32.8	0.14	0.07 to 0.27	< 0.001	87.9	0.25	0.17 to 0.38	< 0.001
Other	84.8	0.79	0.54 to 1.16	0.230	75.4	0.70	0.46 to 1.07	0.101	89.1	0.84	0.57 to 1.24	0.380	29.7	1.16	0.61 to 2.21	0.644	76.3	0.78	0.53 to 1.16	0.219
Can describe IS																				
Yes	81.7	0.13	0.07 to 0.27	< 0.001	73.3	0.14	0.06 to 0.33	< 0.001	81.4	0.14	0.07 to 0.29	< 0.001	77.1	0.23	0.08 to 0.68	< 0.01	79.9	0.16	0.08 to 0.33	< 0.001
No	98.1	1			96.7	1			98.0	1			96.4	1			97.3	1		

*Participants were allowed to select more than one therefore values do not sum to 100%

Note: *IS* Implementation Science; *RCTs* Randomized controlled trials

Compared to academic institutions, research at a public institution was consistently associated with a decreased likelihood of unrecognized IS engagement (aOR 0.54, 95% CI 0.36 to 0.81, *p* < 0.01) including process/implementation evaluations (aOR 0.62, 95% 0.40 to 0.97, *p* = 0.034), use of IS measures (aOR 0.54, 95% CI 0.36 to 0.81, *p* < 0.01), and research integrating an intervention into routine settings (aOR 0.54, 95% CI 0.36 to 0.82, *p* < 0.01).

## Discussion

This study demonstrated the majority of health researchers are aware of IS, with more than two-thirds of the population stating they would be able to describe IS to a colleague; however, comprehensive understanding of IS may not be universal. Despite the high level of self-reported awareness of IS, there may be a general misunderstanding of the scope of IS. An overwhelming majority of health researchers reported at least sometimes using elements of IS; however, when asked directly the type of methods used, only one-tenth of researchers self-identified as using IS. It is not expected that all researchers would or should identify as IS researchers; however, the gap between those identifying as IS researchers and those reporting IS use is larger than would be ideal. The disparity indicates there may be many researchers engaging in IS without being aware their methods would fit under the umbrella of IS research, consider the IS methods used as belonging to another field of research, or do not consistently use a sufficient number of IS methods to consider their work IS. This use of IS elements without identifying them as methods in the field of IS may jeopardize the rigor of the implementation research.

As a field, IS not only seeks to bring attention to the need for real-world relevance in research [14], but, through its frameworks and methods, IS seeks to improve the rigor and transparency of the methods used to examine implementation [1, 14–18]. Many implementation studies in published literature still have weak study designs and lack the rigor necessary to successfully answer important implementation research questions [19, 20]. The potential for the perpetuation of poor practices in implementation research is particularly important as many non-IS health researchers are now expected to incorporate components of implementation into their research [7]. A lack of sufficient awareness of IS methods and training among health researchers could explain some of the shortcomings seen in implementation research. Increasing awareness of IS methods among non-IS researchers who engage in implementation research may lead to more impactful implementation research.

Over the past two decades, considerable progress has been made conceptualizing what constitutes IS [1] and many resources to define and explain IS have been developed [2, 19]. Our study results, however, confirm previous observations that considerable confusion persists about the terminology and scope of IS [18, 21, 22]. The discordance between researchers using elements of IS and those acknowledging the use of IS methods may be partly explained by a confusion regarding what separates IS from other research methodologies. The scope of IS is broad and incorporates many methods and measures familiar to researchers in a variety of other disciplines [1]. Therefore, some health researchers may have been exposed to and using elements of IS as part of research in other fields (e.g., quality improvement).

As many IS resources have been made available only recently, the observed low levels of self-identification as using IS methods may be a result of a lag between IS resource development and dissemination to health researchers. Due to the disconnect between IS element use and the acknowledgement of IS engagement, further efforts are likely needed to disseminate IS to researchers across disciplines. To support these efforts, additional research is needed to determine whether health researchers are aware of and utilizing the currently available IS resources, as well as whether available IS resources provide adequate and sufficiently clear information to be useful for potential IS researchers.

The high prevalence of IS element use reported is at odds with the presentation of these elements in the published literature [23] where publishing even basic IS outcomes are sparse [24–28]. The discordance between using IS methods and what is published in literature may in part be a result of the lack of consistency in IS terminology used. Implementation studies are conducted across a broad range of disciplines and topical areas, and the terminology used to describe similar constructs often varies significantly (e.g., “fidelity” is also reported as “delivered as intended,” “adherence,” “integrity,” and “quality of program delivery”) [23, 29]. Therefore, measuring the use of IS in the literature may underreport the use of these measures. The absence of IS elements in the published literature may also be due to a lack of incentive to publish IS measures, which are often viewed as secondary outcomes for many researchers and publishers alike [30]. Increasing researcher awareness of IS, its methods, and terminology may serve to unify implementation research and increase its impact.

The results of this study support calls for the improvement of researcher training in IS [31–34]. While there are numerous IS resources available [2], it has been acknowledged there is a need for innovative solutions for disseminating such knowledge to researchers [33]. Effective training in IS is essential for the success of IS research [31, 32], and the dissemination of IS knowledge may reduce unrecognized IS engagement and consequently improve the effectiveness and impact of implementation research.

### Limitations

Our study had several limitations. First, the generalizability of our study may be limited due to selection bias from the sampling frame used. NIH RePORTER is limited to researchers who have had a successful grant submission. Therefore, the survey data may not be generalizable to researchers using other non-public sources of funding, more junior researchers, or those who have been unsuccessful in getting funding. Similarly, NIH RePORTER predominantly contains USA researchers and therefore the study results may not be generalizable to researchers outside of the USA. Second, this study was likely impacted by response bias due to the nature of the survey topic. The survey invitation purposefully did not include terms associated with IS and as a result, approximately one-quarter of researchers who started the survey did not complete it, with a number of researchers expressing (through personal correspondence with the author) frustration and disinterest in completing the survey because it was not relevant to them or their research. Therefore, it is likely greater survey completion was present in researchers who were already aware of and engaging in IS. Similarly, the overall response rate was relatively low and therefore the estimates reported may not be representative of the sampling frame as a whole. However, overall, the distribution and variety of reported methods used indicate that the group that completed the survey still represents a diverse group of health researchers that are likely to be generally representative of the target population [13]. Finally, while the pilot tested, the survey measures of IS engagement have not been validated. Our results are also based on self-report of elements of IS and not actual practice or understanding of IS, which is likely to lead to an overestimation of the number of researchers engaging in implementation research. Additionally, the survey did not measure the quality of research being performed by those with unrecognized IS and more research is needed to assess actual IS practices in this population.

## Conclusions

Overall, awareness of IS is high among health researchers, yet there is also a high prevalence of unrecognized IS engagement. Efforts need to be made to further disseminate what constitutes IS research and increase IS awareness among health researchers.

## Data Availability

The datasets used and/or analyzed during the current study are available from the corresponding author on reasonable request.

## Abbreviations

IS: Implementation science
aOR: Adjusted odds ratio
95%CI: 95% confidence interval
