# Correction to: Unrecognized implementation science engagement among health researchers in the USA: a national survey

**DOI:** 10.1186/s43058-020-00056-y

**Published:** 2020-07-15

**Authors:** Elizabeth R. Stevens, Donna Shelley, Bernadette Boden-Albala

**Affiliations:** 1 Department of Population Health, NYU Langone Health, New York, NY USA; 2 College of Global Public Health, NYU, New York, NY USA; 3 Susan and Henry Samueli College of Health Sciences, UC Irvine, Irvine, CA USA

**Correction to: Implementation Science Communications (2020) 1:39**

**https://doi.org/10.1186/s43058-020-00027-3**

Following publication of the original article [1], it was reported that a supplemental appendix (Additional file 1) was missing. The missing appendix is included in this Correction article.

## Supplementary information

**Additional file 1.** Relevant analysis survey questions.
