# Supplementary material for: Correction to: Unrecognized implementation science engagement among health researchers in the USA: a national survey
Source: Implement Sci Commun. 2020 Jul 15;1:65. doi: 10.1186/s43058-020-00056-y (PMC7427891; doi:10.1186/s43058-020-00056-y)

**Appendix 1: Relevant analysis survey questions**


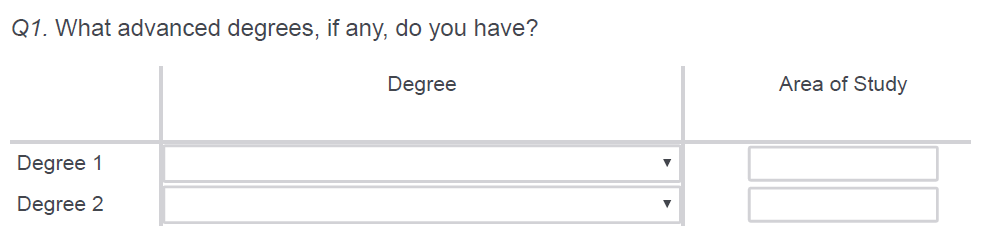


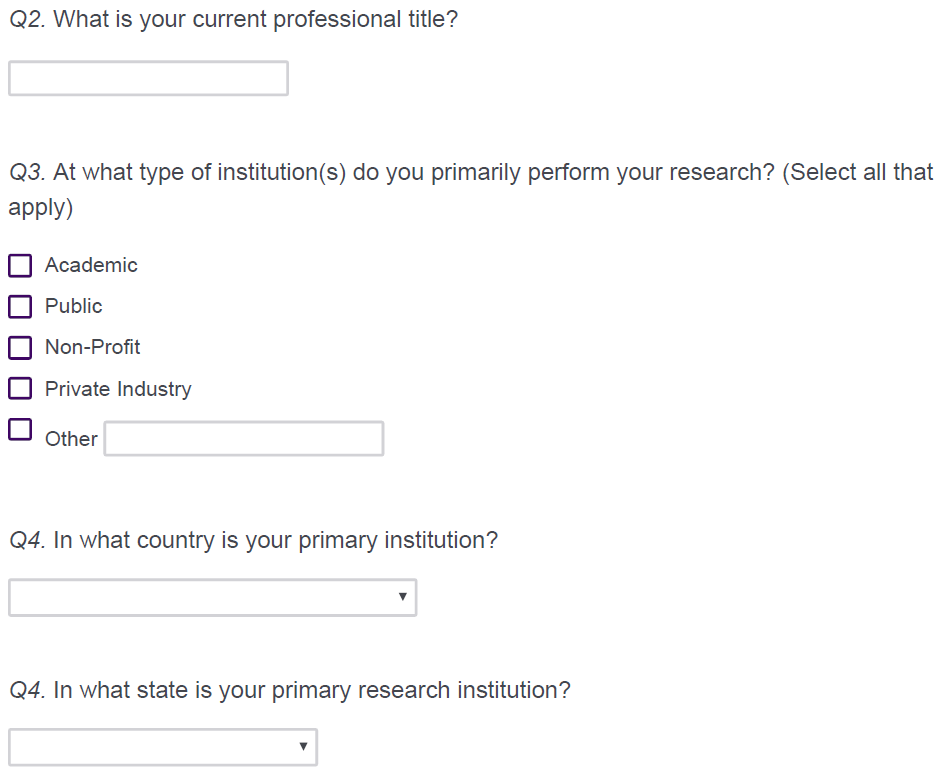

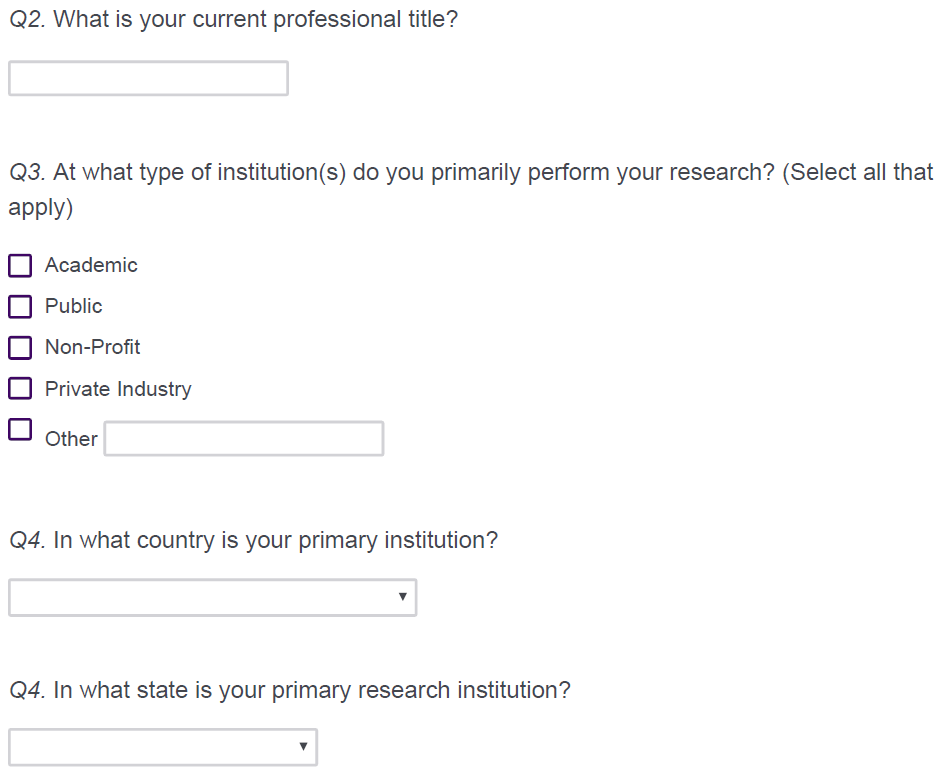


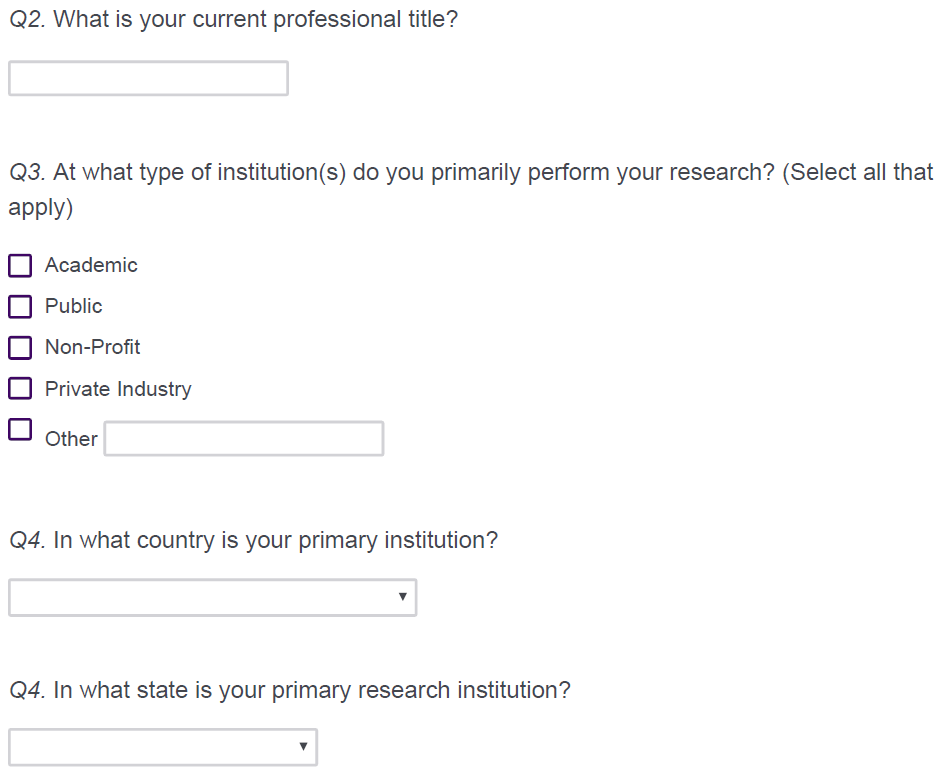


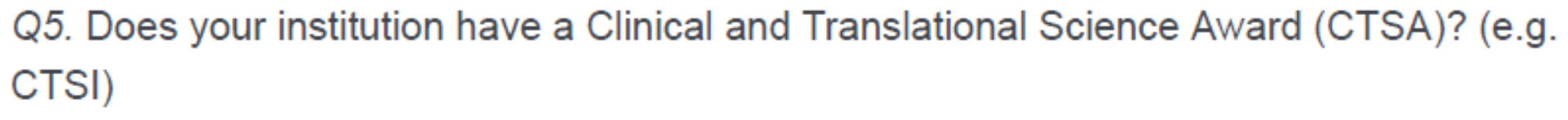


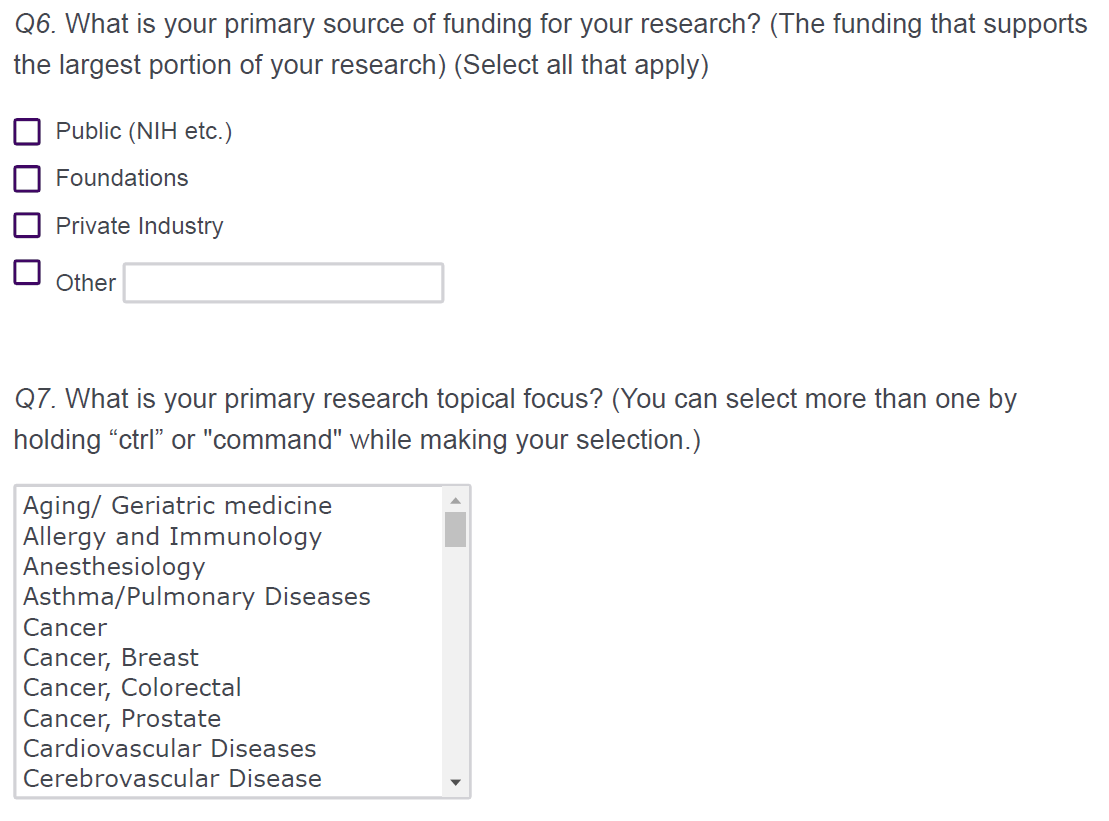

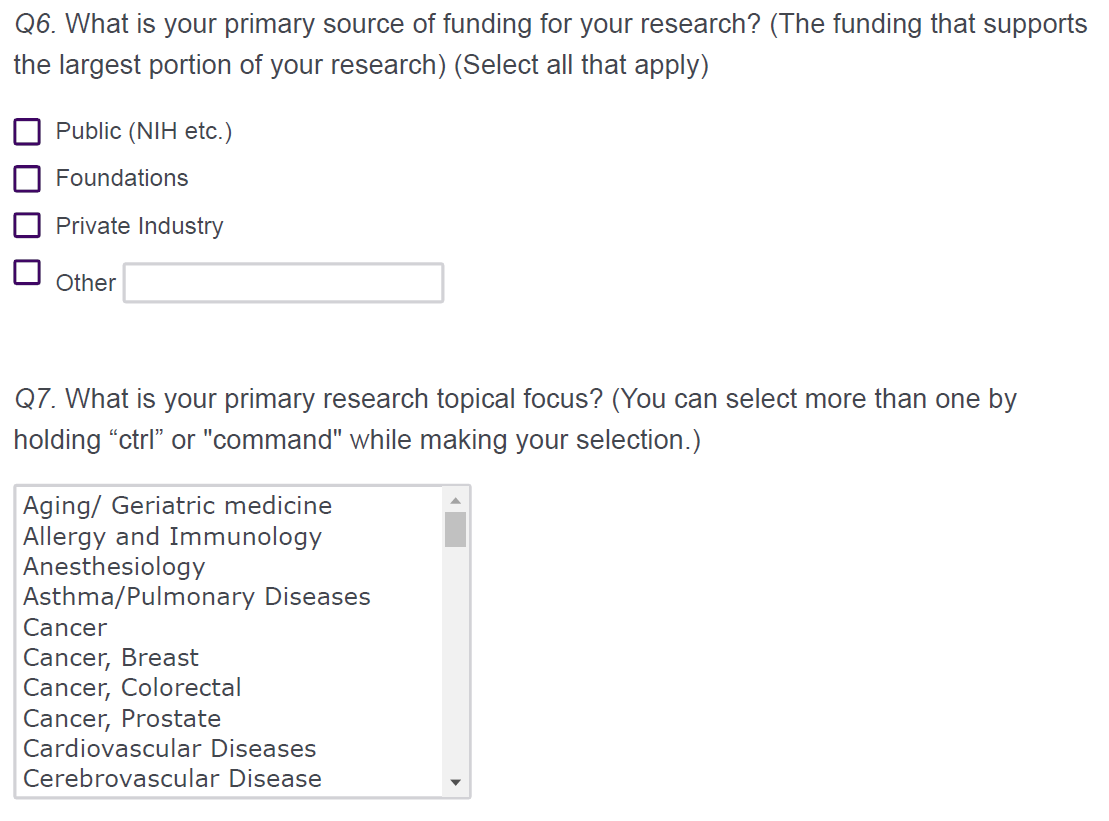


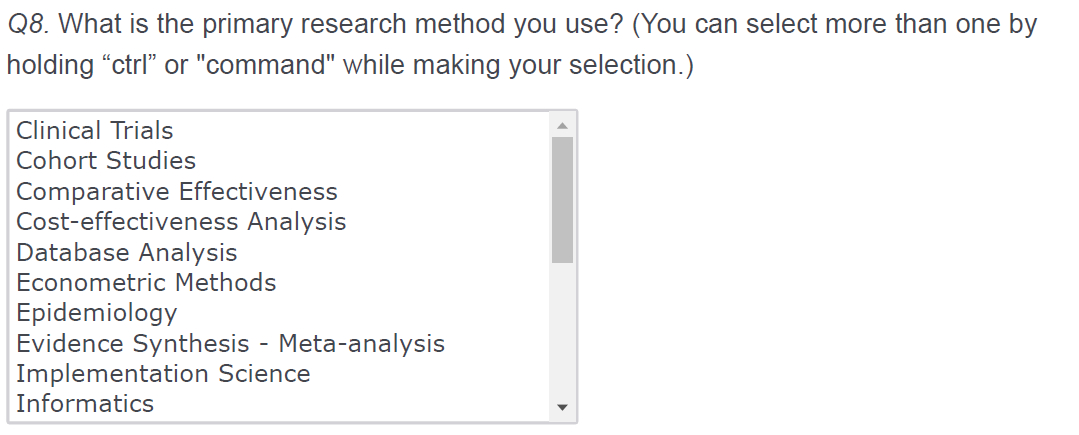


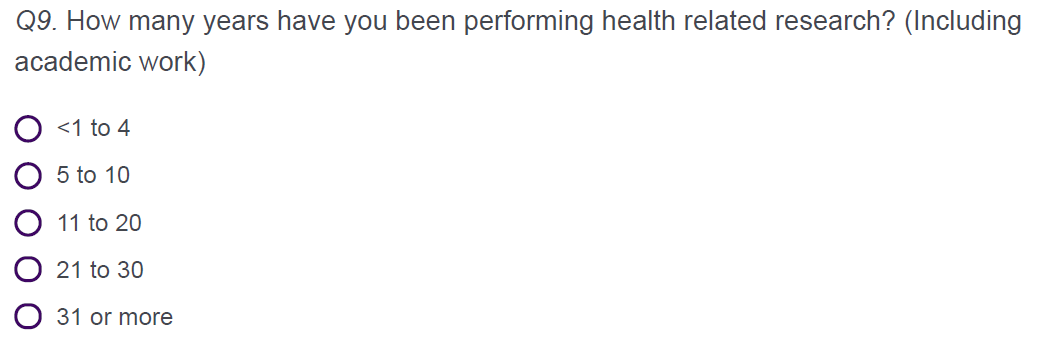

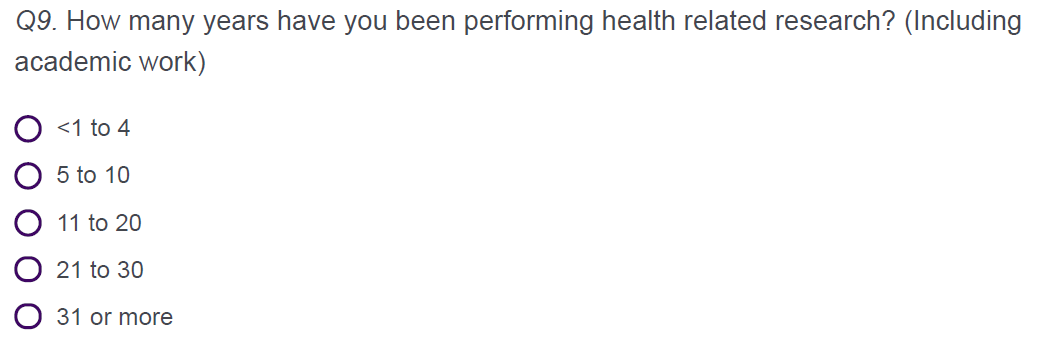

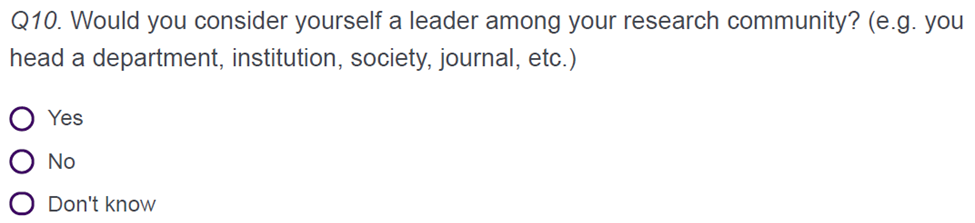

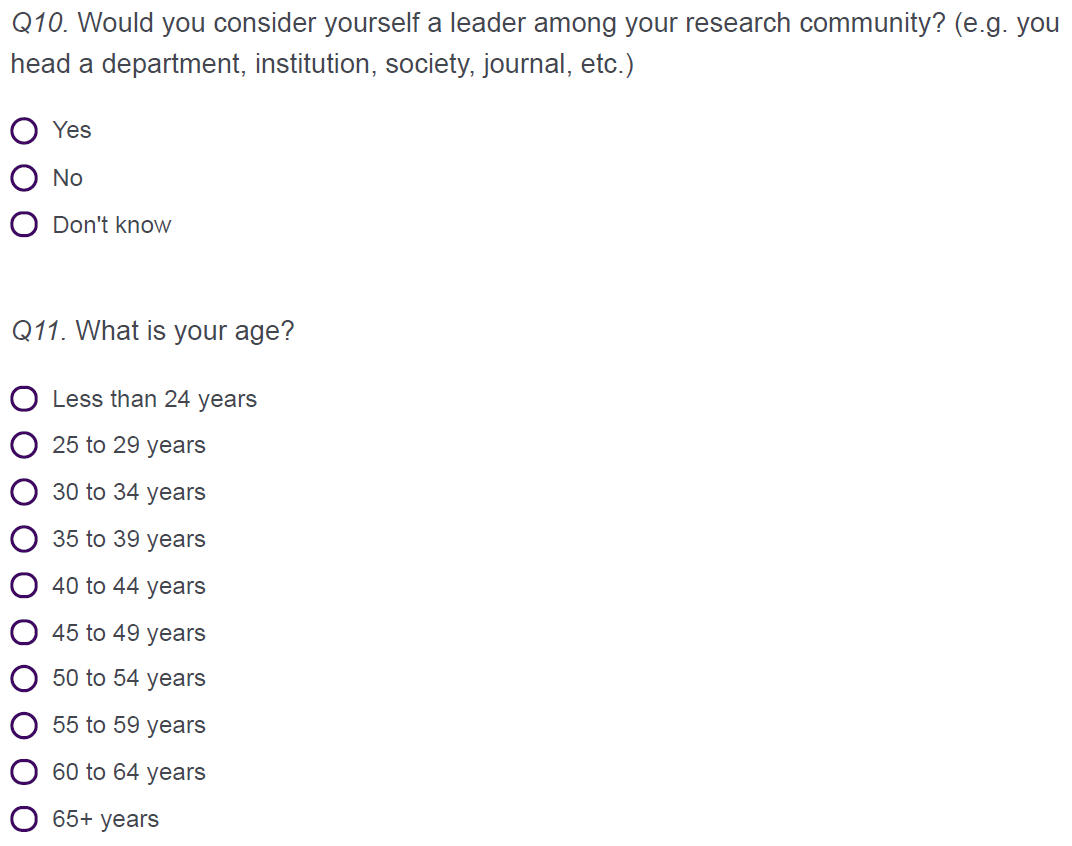


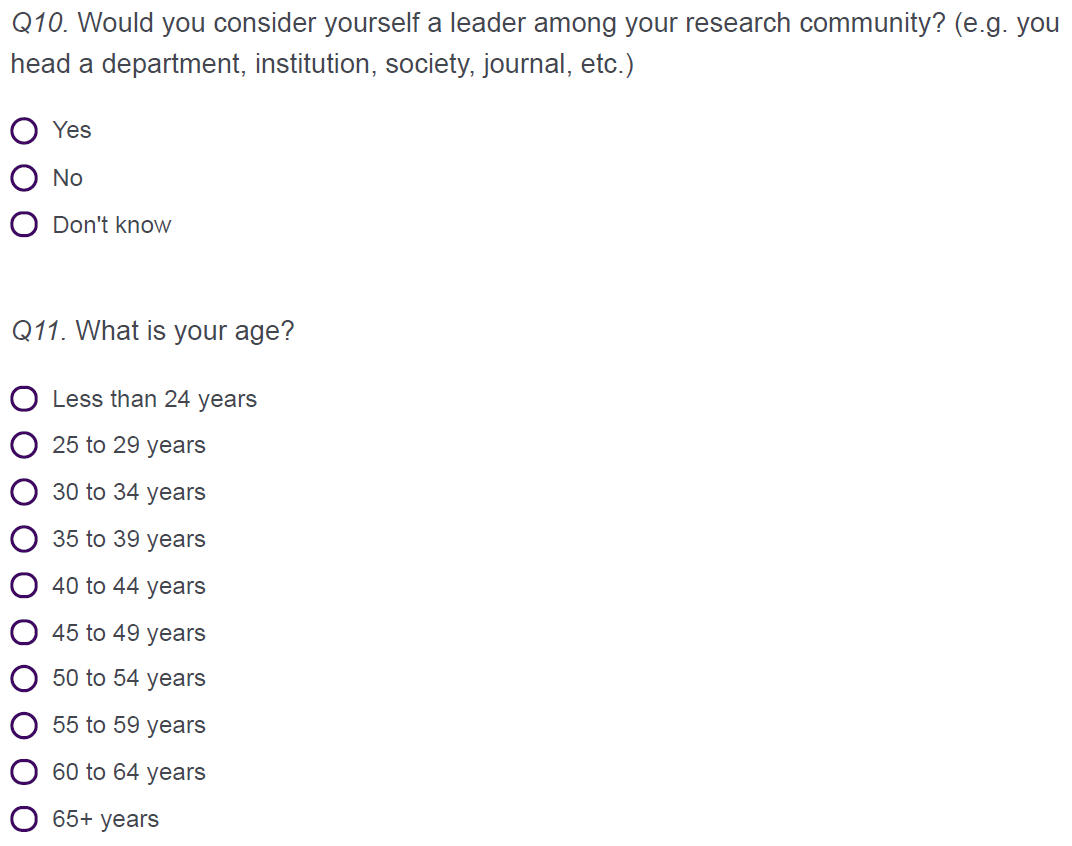


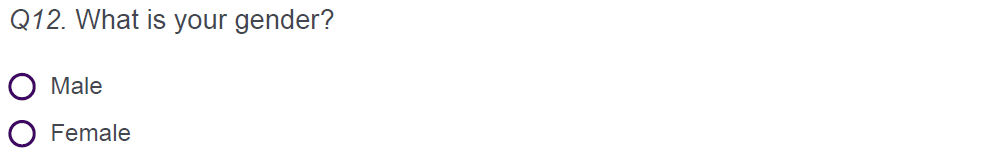


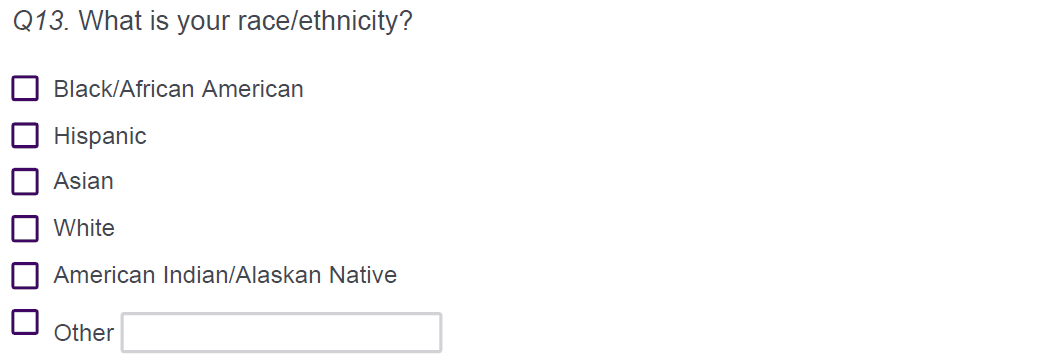


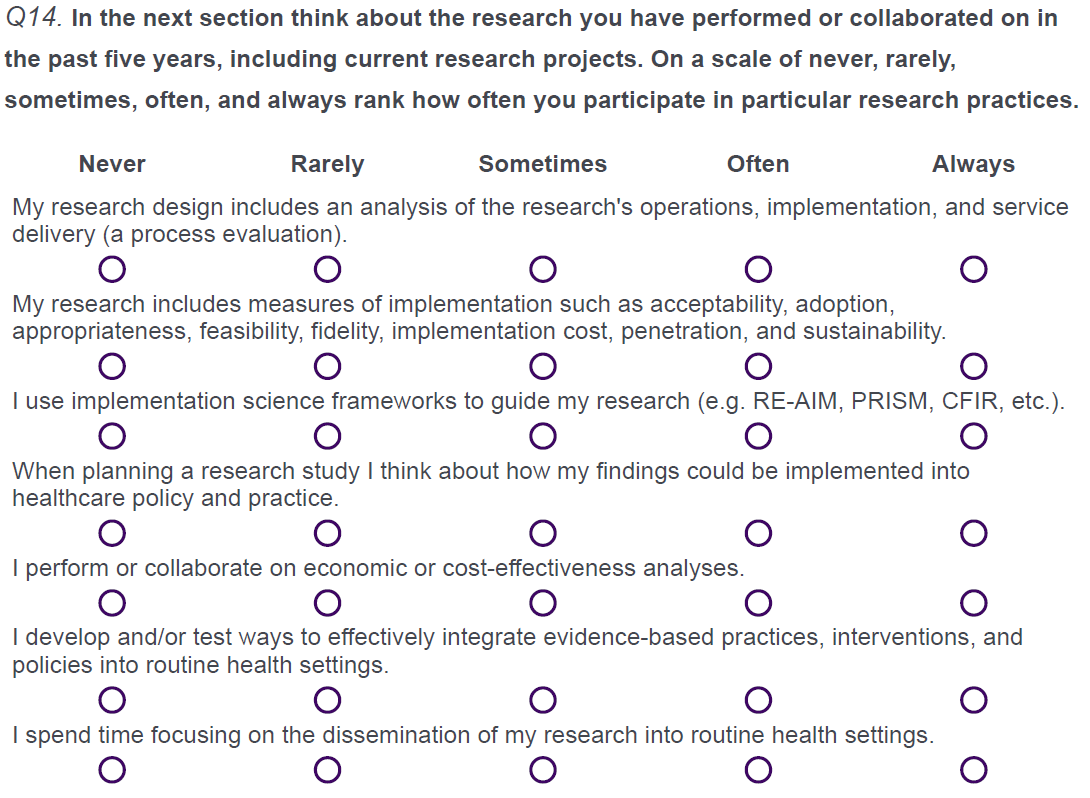


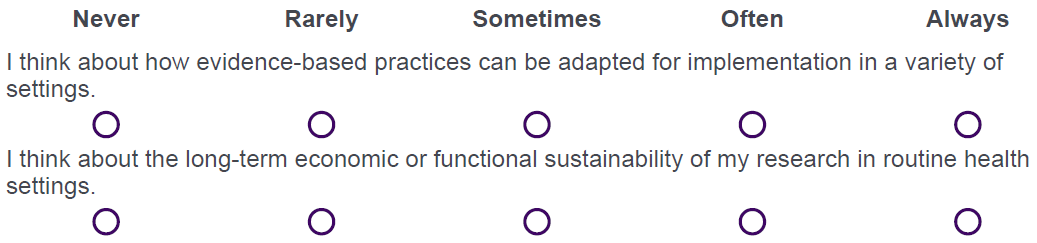


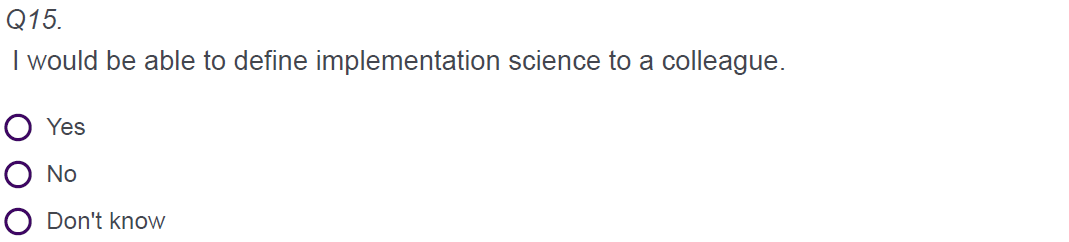


IMPLEMENATION SCIENCE DEFINED AFTER ABOVE QUESTIONS


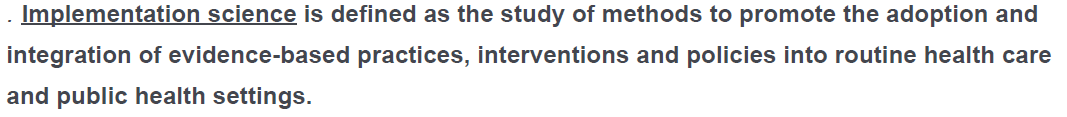


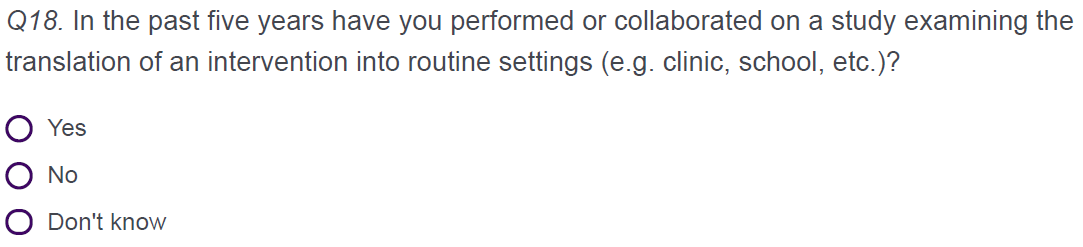

Supplement: Supplementary file 1 — Additional file 1. Relevant analysis survey questions. [file 43058_2020_56_MOESM1_ESM.docx]
